# Enhanced Signed Graph Neural Network with Node Polarity

**DOI:** 10.3390/e25010038

**Published:** 2022-12-25

**Authors:** Jiawang Chen, Zhi Qiao, Jun Yan, Zhenqiang Wu

**Affiliations:** School of Computer Science, Shaanxi Normal University, Xi’an 710062, China

**Keywords:** signed network, network embedding, graph neural network, node polarity

## Abstract

Signed graph neural networks learn low-dimensional representations for nodes in signed networks with positive and negative links, which helps with many downstream tasks like link prediction. However, most existing signed graph neural networks ignore individual characteristics of nodes and thus limit the ability to learn the underlying structure of real signed graphs. To address this limitation, a deep graph neural network framework SiNP to learn Signed network embedding with Node Polarity is proposed. To be more explicit, a node-signed property metric mechanism is developed to encode the individual characteristics of the nodes. In addition, a graph convolution layer is added so that both positive and negative information from neighboring nodes can be combined. The final embedding of nodes is produced by concatenating the outcomes of these two portions. Finally, extensive experiments have been conducted on four significant real-world signed network datasets to demonstrate the efficiency and superiority of the proposed method in comparison to the state-of-the-art.

## 1. Introduction

With the explosive growth of online media in recent years, the social network has become an essential area of connecting and better understanding online human behavior by examining the user’s online transactions. In particular, signed networks, which contain positive (like trust, approval, and support) and negative (like distrust, disapproval, and disagreement) relationships on the connected edges of nodes and assist users in discovering complex interactions from the social network, play a key role in several online social media sites [1]. There has been a significant amount of research conducted in this field due to the fact that these interactions are human-created and influence people’s opinions and perspectives in their social lives. Earlier social psychologists have been devoted to the development of signed network analysis based on social theories such as the balance theory [2]. In signed network analysis, signed link prediction is used to predict the positive or negative relationships on the connected edges that exist in a variety of social media. Trust network are one example, in these networks, people are represented by nodes, and the positive and negative links that connect the nodes serve as a model for trust and distrust relations [3].

**Figure 1:**
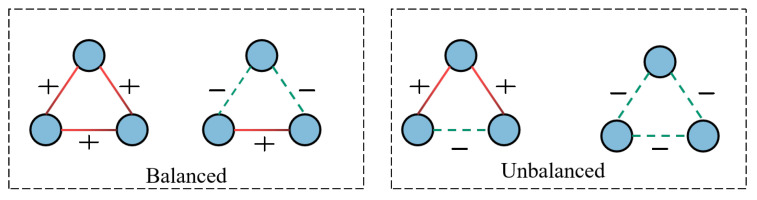
Balance and unbalance relations in a signed network.

Several studies have been conducted to solve the problems of predicting signed links [4,5,6,7], some earlier approaches adopted normalized spectral analyses model, log-bilinear model and so on. Although the approaches have successfully achieved signed link prediction, they have some drawbacks. To begin, they are very dependent on features that have been manually constructed and do not perform well when applied to real-world settings. Moreover, they ignore that signed networks carry special properties on their edges. To address these flaws, studies on automatic feature extractions for signed link prediction [8,9,10,11] have been conducted that aims to learn fixed-length vector representations for each node by constraining the node’s proximity. In particular, several studies adopted graph neural networks to mine the potential information from the signed networks because it is the most powerful technique for identifying the representation required from a large data set, replacing manual engineering, and allowing the systems to both understand and use the features to perform tasks such as community detection [12], node classification [13] and link prediction [14,15,16]. These techniques modeling signed network follow message passing neural networks with social theory constraints (such as the balance theory). According to the social theories, nodes in the signed network with positive relations are embedded near together, whereas nodes with negative relations are embedded far apart. Figure 1 provides a straightforward summary of the balance theory. The two triangles on the left of Figure 1 are structural balance triangles. In contrast, in the unbalanced structural triangles on the right, the relationship between a pair of nodes is both friend and foe. SGNNs have been shown to achieve promising results on many signed graph analytic tasks. However, none of the existing methods take the individual characteristics of the nodes into consideration, which is critical for improving the representation power of a graph. This is because individual characteristics may transmit potential attribute information of nodes to guide the representation learning process.

Motivated by these limitations, we propose SiNP, a novel polarity encoding-based signed graph neural network framework to learn signed network embedding with individual characteristics. Instead of only applying aggregators when aggregating information from neighboring nodes, SiNP employs a signed property metric mechanism to learn the potential attributes information of real signed graphs and this information is then used to drive the process of feature aggregation for surrounding nodes. The following is a synopsis of the most important contributions made by this article:

- First, we introduce a node signed property metric mechanism to effectively learn signed network embedding, which utilizes a node polarity metric mechanism to learn the potential attributes information of nodes.
- Next, we design an objective function for both framework optimization and node representation learning, which includes the objectives of edge signed classification and structural balance theory.
- Finally, we conduct extensive experiments on four real-world signed network datasets to validate the efficacy of the proposed SiNP framework through the signed link prediction task.

The structure of this paper continues below. Section 2 reviews signed network embedding and graph neural network studies. Section 3 outlines the SiNP framework a priori information. Section 4 analyzes the method. Section 5 concludes the paper.

## 2. Related Work

### 2.1. Signed Network Embedding

A plethora of signed network embedding techniques have been presented in recent years as a solution to the problem that traditional unsigned network emebdding methods fail to adequately convey the unique semantic information conveyed by positive and negative edge representations [4,5,6,7,8,9,10,11]. To produce the low dimensional representation of nodes, these techniques combine standard machine learning procedures with the specialized sociological theory of signed networks, such as balancing theory. To address the issue of negative connection prediction in signed networks, Tang et al. [10] suggested a NeLp technique that makes use of the soft edge support vector. Wang et al. [9] validated the status theory of users in trust relationships and computed the status of users in social networks by using the PageRank algorithm. In their approach, the prediction of trust relationships between users was achieved by using non-negative matrix decomposition combined with user status theory. In [11], Yuan et al. proposed SNE, a signed network node representation learning algorithm that employs a random walk strategy to generate a sequence of nodes. The algorithm of SiNE [5] utilized a multilayer neural network for learning the embedding representation of nodes without using softmax as well as log-likelihood, by maximizing the probability of node co-occurrence. SiNE designs a refined strategy based on triangle relations to extract similarities and dissimilarities between nodes, which efficiently and accurately extracts the structural properties of the network.

Although they address the limitations of the existing traditional network representation learning methods, the available signed network embedding methods cannot processing data from end-to-end.

### 2.2. Graph Neural Network

The goal of graph neural networks (GNNs) is to bring the power of deep learning to structured datasets. Regular deep neural networks, such as CNNs, are not applicable in graph domains because of the non-Euclidean nature of the data [17,18]. To solve this problem, GNNs ignore the input order of nodes and propagate the information on each node separately. They can also do the propagation guided by graph structure instead of using it as part of node features. It has an advantage because it is the most powerful technique for identifying the representation required from a large network dataset, replacing manual enfineering and allowing the system to both understand and use the features to perform tasks. As a classic graph neural network, a graph convolution network (GCN) [19] uses feature decomposition and Fourier transform with the help of the Laplacian matrix to obtain a convolution kernel to perform convolution on the graph network. GraphSage [20] is an inductive framework that can use vertex feature information such as text attributes to efficiently generate never before seen embedding for vertices. GAT [21] assumes that neighbor nodes have different contributions to the central node. An attention mechanism is used to learn the significance of each neighbor node to the center node. With the continuous research on graph convolution, researchers have also proposed some signed network embedding methods based on deep graph convolution networks. Signed Graph Convolutional Network (SGCN) [14] first expanded GCN to signed networks and designed both positive and negative aggregators to generate “friend expression” and “enemy expression” for each node in signed networks based on balance theory. SiGAT [22] introduces an attention mechanism to signed-directed networks and develops a social theory-based graph neural network model. Similarly, SNEA [23] proposed a graph attention layer and offers a more generalized strategy for aggregating data via positive and negative links in accordance with balancing theory.

The major limitation of the existing graph neural network-based embedding approaches is that they ignore the individual characteristics of the nodes, which significantly limits their ability to learn the underlying structure of real signed graphs. In contrast, our proposed SiNP method employs a node property metric mechanism to learn the potential attributes information of real signed graphs and use the learned information to guide the feature aggregation process for neighboring nodes.

## 3. The Proposed SiNP Model

Before describing the SiNP in detail, we explain how signed GNNs work. Signed graph neural networks are deep neural networks with feature learning. Negative edges in signed graphs have distinct physical meanings than positive ones, making convolutional processes different. However, the vast majority of signed graph neural networks do not take into account the correlation between nodes during feature aggregation, which in turn reduces the effectiveness of multiple downstream tasks (e.g., link prediction between node pairs). Actually, the correlation between nodes (e.g., personalized features) is crucial for feature learning in signed graphs. Conventional graph metrics for node similarity (e.g., common neighbors) only consider the number of neighbors and ignore their type, thus failing to accurately characterize the correlation between nodes in a signed graph. This study identifies, for the first time, the limitations of the representational power of existing signed graph neural networks and analyses the effectiveness of relevance modeling in enhancing their representational power. Subsequently, a node correlation metric is defined to measure the distance between nodes and a signed distance encoding mechanism is designed to encode the relevant information. Based on this, a signed graph neural network based on polarity distance coding is designed, which takes into account the relative distances between nodes when aggregating information from neighbors.

### 3.1. Related Definitions

In this subsection, we introduce a few primary terms and notations used in this paper to simplify the presentations. Given a signed network G = (V,E), which is constructed of a set $V=\left\{v\_1,v_{2},\ldots ,v_{N}\right\}$ with *N* nodes, the set of positive links between nodes can be expressed as $E^{+}$ and the set of negative links can be denoted as $E^{-}$. Note that $E=E^{+}\cup E^{-}\mathrm{and}\;E^{+}\cap E^{-}=Ø$. The set of positive and negative neighbors of a node can be expressed as $N_{i}^{+}\mathrm{and}\;N_{i}^{-}$, respectively.

### 3.2. Signed-Distance Encoding Module

**Figure 2:**
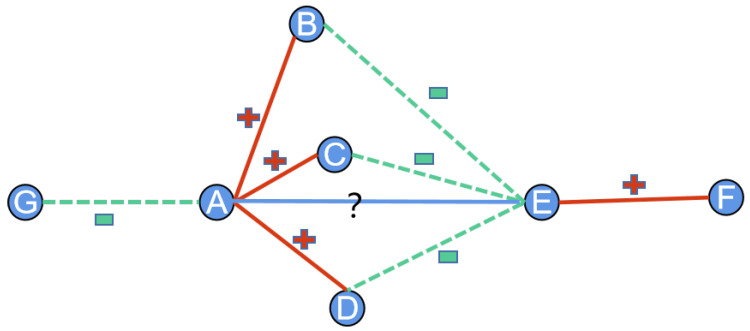
An example of the signed distance module.

One of the easiest ways to model the relative distance between nodes on a signed graph is the number of common neighbors. However, common neighbors focus only on the number of identical neighbors, ignoring the effect of neighbors with different relationships on relative distance. This leads to sub-optimal performance in subsequent signed graph analysis. Take nodes *A* and *B* in Figure 2 as an example, both of them have three common neighbors, but in the signed network, according to the balance theory, node *A* and node *B* prefer a negative relationship to achieve structural balance. By calculating and encoding the polarity information of the nodes, the nodes’ own unique neighbor relationships can be encoded into the initial features to better guide the downstream tasks of signed graph analysis.

For a node in the signed network, let D(V) = $d^{-}\left(v\right)$ + $d^{-}\left(v\right)$ be the degree of node *v*, we define the signed polarity of each node as follows:

(1) $$p^{-}\left(v\right)=-\ln\frac{d^{-}\left(v\right)}{D(V)},\;p^{+}\left(v\right)=-\ln\frac{d^{+}\left(v\right)}{D(V)}$$

where $p^{-}\left(v\right)$ denote negative signed polarity and $p^{+}\left(v\right)$ denote positive polarity.

Then, the signed polarity-based distance between node *i* and node *j* can be defined as:

(2) $$\gamma_{ij}=\left|\right|\left({p_{i}}^{-}\left(v\right),\;{p_{i}}^{+}\left(v\right)\right),\left({p_{j}}^{-}\left(v\right),\;{p_{j}}^{+}\left(v\right)\right){\left|\right|}_{2}^{F}$$

We call the n*n matrix M = [*ij*] the signed polarity-based similarity matrix.

Based on signed polarity distance, signed distance coding can further encode and model the correlation between the set of the target nodes and individual nodes as follows:

(3) $$h_{i}^{dis}=F\left(E\left(\gamma_{ij}\right)\right)$$

where *F* denotes the fusion function, such as the sum operation, *E* denotes the encoding function like the one hot encoding. Given the node pair (*A*,*B*) to be predicted, the signed polarity distance of node *i* with respect to the node pair (*A*,*B*) is given as follows:

(4) $$h_{i}^{A,B}=F\left(E\left(\gamma_{iA}\right),E\left(\gamma_{iB}\right)\right)$$

In addition to the node pair (*A*,*B*), the set of target nodes can also be individual nodes or even the whole graph. *A* deeper study of the signed polarity distance of node *i* with respect to (*A*,*B*) reveals that: node *i* plays a bridging role in the signed link prediction between *A* and *B*, and also measures the relative distance of nodes pairs (*A*,*B*). Capturing the relative distance information plays a crucial role in the signed link prediction task.

Finally, the signed distance encoding of the nodes is linearly transformed through the fully connected neural network, and the initial representation of node *i* can be obtained as shown below:

(5) $$h_{i}^{0}=\sigma \left(W_{0}\cdot h_{i}^{dis}+b_{0}\right)$$

where σ represents the activation function, and $W_{0},b_{0}$ represents the weight matrix and bias vector, respectively. We project the original signed distance information as a learnable vector. This significantly increases its representation capacity and allows for end-to-end optimization and learning based on back propagation.

### 3.3. Signed Convolution Module

Message-passing neural networks (MPNNs) are an universal class that can be used to the vast majority of graph neural networks. The definition of an aggregation function and an update function between nodes is at the heart of multi-processor neural networks (MPNNs) [24]. To get started, the local structural expression of each node is determined by first applying the aggregation function to that node as well as the nodes that are located in its immediate neighborhood. Second, the current node’s representation is updated using the update function and the local structural representation. The general expression of the MPNNs can be expressed as

(6) $$m_{i}^{t+1}=\sum_{j\in N(i)}M_{t}\left(h_{i}^{t},h_{j}^{t},e_{i,j}\right),h_{i}^{t+1}=U_{t}\left(h_{i}^{t},m_{i}^{t+1}\right),$$

where $h_{i}^{t}$ denotes the hidden layer representation of node *i* at t-th steps, $e_{i,j}$ is the features of a given link, $M_{t}$ represents the aggregate function at t-th steps, $m_{i}^{t+1}$ means the local structure representation of node *i* after aggregating, and $U_{t}$ stands for the update function. By designing appropriate sampling and aggregation functions, such as weighted aggregation or mean aggregation, the target node accepts the features passed from its own neighbourhood nodes and completes an update of its own features through feature fusion of the local structure to obtain a new feature representation.

Signed network is a specialized type of network that contains type information on its edges. It not only includes two types of connected edges (positive links and negative links), but also has special sociological properties such as structural balance. In particular, the fact that my enemy’s enemy is my friend (a foe node two hops from the central node is a friend) makes it infeasible to define aggregation functions only based on edge type. As a result, we use two different GNN aggregators in this paper to aggregate different information from $N_{i}^{+}$ and $N_{i}^{-}$.

In the first aggregation layer, given the initial feature $h_{i}^{\left(0\right)}$ of node *i*, we can generate the balanced embedding $h_{p}$ and unbalanced embedding $h_{n}$:

(7) $$h_{p}^{B(1)}=\sigma \left(\sum_{j\in {\hat{N}}_{i}^{+}}{\tilde{F}}_{agg}h_{i}^{\left(0\right)}h_{j}^{\left(0\right)},W^{B(1)}\right),$$

(8) $$h_{n}^{U(1)}=\sigma \left(\sum_{j\in {\hat{N}}_{i}^{-}}{\tilde{F}}_{agg}h_{i}^{\left(0\right)}h_{j}^{\left(0\right)},W^{U(1)}\right),$$

where σ() is the nonlinear activation function, ${\tilde{F}}_{agg}$ is the aggregate operation for aggregating feature information from node pairs, $W^{B(1)},W^{U(1)}\in R^{d_{in}^{\left(1\right)}\times d_{out\;}^{\left(1\right)}}$ refers to the linear transformation matrices responsible for the information aggregated from $N_{i}^{+}$ and $N_{i}^{-}$, and $d_{out}$ denotes the length of hidden embeddings. Due to the fact that the first layer of the model can only portray first-order neighbors, there is no structural balance and friends or enemies can be obtained by direct aggregation. However, from the second layer of the model, the friend representation of node *i* will be acquired by the aggregator from its own friends, its own friends’ friends and its own enemies’ enemies based on balance theory.

For the deeper aggregation layers (l>1), it can be recursively defined as

(9) $$\begin{array}{c} h_{p}^{B(l)}=\sigma (\sum_{j\in {\hat{N}}_{i}^{+},k\in N_{i}^{-}}{\tilde{y}}_{ij}^{B(l)}h_{j}^{B(l-1)}W^{B(l)}\;\\ +{\tilde{y}}_{ik}^{B(l)}h_{k}^{U(l-1)}W^{B(l)}) \end{array},$$

(10) $$\begin{array}{c} h_{n}^{U(l)}=\sigma (\sum_{j\in {\hat{N}}_{i}^{+},k\in N_{i}^{-}}{\tilde{y}}_{ij}^{U(l)}h_{j}^{U(l-1)}W^{U(l)}\;\\ \;+{\tilde{y}}_{ik}^{U(l)}h_{k}^{B(l-1)}W^{U(l)}) \end{array},$$

where $W^{B(l)}$, $W^{U(l)}$ is the shared weight matrix. When the number of aggregation layers is greater than two, the balanced embedding of node $v_{i}$ should not only aggregate the information from the balanced node set, but also nodes from the unbalanced node set, whose relationship is enemy’s enemy.

### 3.4. Objective Function and Training

In this section, we discuss the objective function as well as the training details of proposed SiNP. Considering that there are two types of connections in signed networks: positive links and negative links, which are all represented by S={+,−}. In the hidden space, we reduce the distance between positive node pairs as much as possible while increasing the distance between negative node pairs. *A* binary edge classification problem is derived from the original optimization problem. The binary cross-entropy (BCE) loss is therefore used as follows:

(11) $$L_{\mathrm{loss}(o,\;t)}=-1/n\sum_{i}\left(t\left[i\right]*\log\left(o\left[i\right]\right)+\left(1-t\left[i\right]\right)*\log\left(1-o\left[i\right]\right)\right)$$

where *n* stands for the number of training data, t[i] denotes the true label of the training data, and o[i] denotes the output of the model, which can be obtained by an MLP predictor:

(12) $$o\left[i\right]=Sigmoid(MLP\left(\left[Z_{u},Z_{v}\right]\right))$$

The overall objective function L can be defined as

(13) $$L=L_{\mathrm{loss}(o,\;t)}+\lambda L_{\mathrm{reg}},$$

## 4. Experiment and Analysis

Here, we describe in detail the experimental methods we employ to assess the efficacy of the proposed SiNP framework. The evaluation metrics, state-of-the-art baseline model, and data description and preprocessing are all part of this. We then evaluate the quality of the learnt node embeddings and give a sensitivity analysis of the suggested SiNP’s parameters. We conclude by contrasting the efficiency of the proposed SiNP with that of the standard algorithms.

### 4.1. Data Description and Pretreatments

We used four well-known signed network datasets—Bitcoin-Alpha, Bitcoin-OTC, Slashdot, and Epinions—to assess the effectiveness of the proposed SiNP framework. Both Bitcoin-Alpha and Bitcoin-OTC are user-user trust/distrust cryptocurrency networks that allow users to trade anonymously over the web using platforms and accept Bitcoin as a payment option [25]. Based on these two datasets, users’ preferences can either distrust or trust others on a scale of −10 to +10. *A* score less than 0 is regarded as negative, while a score greater than 0 is regarded as positive. Slashdot [26] was launched in February 2009, with 79k nodes and 723k edges. The Zoo features in the dataset let users distinguish friends and opponents. As a technology news website, visitors can contribute and read editor-approved content. In the Epinion dataset, users can score a review’s helpfulness from 1–5. The edges between the two users indicate that at least one review of the other user was helpful. The helpfulness score is translated to the interval [−1, +1], where helpfulness scores (positive and negative) of 1, 2, 3, 4, and 5 are scaled to −1.0, −0.5, 0.0, 0.5, and 1.0, respectively, and the weight of the edge is the average of the multiple helpfulness scores from one user to other. The datasets used to evaluate the effectiveness of the proposed SiNP are similar, and Table 1 provides a detailed summary. The test data for each dataset is set at 20%,while the training data is set at the remaining 80%.

### 4.2. Baseline Methods

In experiments, four state-of-the-art baseline methods are adopted for comparison to show the superiority of the proposed method, including two signed network embedding methods (SiNE, SIDE) and two signed graph neural network methods (SGCN, SNEA).

- SiNE [5] creates network embeddings using a deep learning framework by optimizing the objective function in a signed network while being guided by the balance theory.
- Sign2vec [7] uses targeted node samplings for random walks to maintain the structural balance using high-order neighborhoods.
- SGCN [14] embed nodes in a network using balancing theory by developing a two-node aggregator and sharing data with a GCN.
- SNEA [23] generated embedding for nodes using a balance-theory-based metric that uses a self-attention process to estimate the coefficient of importance for each pair of nodes.

### 4.3. Parameter Settings

SiNP is implemented on the Pytorch, with the model parameters initialised to a Gaussian distribution and updated using Adam. During the experiments, the total number of training epochs used is 500, the length of the signed path is set to 10, the learning rate is initiated to be 0.001, the regularization factor λ is set to 0.001. For the path-level attention, the dimension is set to 128, the number of attention heads *k* is set to 8, and the final embedding dimension is set to 64. We represent the features of the links as the union of two-node embedding representations. Consequently, the signed link prediction problem can be transformed into a binary classification problem i.e., classifying positive or negative links. For that, we select the logistic regression task as a binary classifier similar to the existing baseline methods [14,23], and also the node embedding is initialized with TSVD.

On each of the datasets, we compared the proposed SiNP to baseline models using a variety of performance metrics, including: Area Under the Curve (AUC), the micro-average F1-score (Micro-F1), the macro-average F1-score (Macro-F1), and the binary-average F1-score (Binary-F1). When measuring the effectiveness of the proposed SiNP framework, the measures employed are comparable to those employed by the foundational models. Both the metrics access the quality of signed link prediction accuracy especially when the positive ad negative links are imbalanced.

### 4.4. Performance Comparison with Baseline Methods

The performance of the SiNP proposed against baseline approaches is compared first. Table 2 shows the performance comparison with the best performance. The following can be observed:

- Sign2vec and SiNE show the worst AUC performance on three datasets. This shows that the conventional methods are not convenient for signed link predictions. The SGCN, SNEA outperform the Sign2vec and SiNE on all four datasets, and the proposed SiNP, outperforms all the existing baseline methods for all the signed network datasets with an improved AUC of 5%, demonstrating the emphasis of the proposed SiNP by applying balance theory as well as the individual characteristics of nodes.
- SiNP significantly outperforms all the baseline models on Micro-F1, Macro-F1, and Binary-F1. In particular, on the Bitcoin-Alpha dataset, SiNP outperforms all the best baselines by 0.928, 0.791, and 0.959 on Micro-F1, Macro-F1, and Binary-F1, respectively. On the Bitcoin-OTC dataset, SiNP outperforms all the best baselines by 0.920, 0.835, and 0.950 on Micro-F1, Macro-F1, and Binary-F1, respectively. SiNP outperforms all the baseline models on Slashdot dataset. It performs the best baseline by 0.836, 0.771, and 0.894 on Micro-F1, Macro-F1, and Binary-F1, respectively. We can observe from Table 2 on epinion datasets, SiNP outperforms the best baseline by 0.903 and 0.941 Micro-F1, and Binary-F1, respectively, but, SiNP perform better than the baseline model with Macro-F1 of 0.819. This shows that considering node-level and path-level node representation can significantly improve the sign link prediction outcomes.
- The finding shows that the use of GNN models has strong capabilities over the existing signed network embedding techniques. As shown in Table 2, SNEA performs the best among the two balance theory-based SGNNs, demonstrating that fine-grained mining of the network structure can effectively improve model performance. However, SiNP showed a significant performance improvement over MUSE over AUC, Micro-F1, and Binary-F1, reflecting the limitations of the simple consideration of node features.

**Table 2:** The results of signed link prediction on four datasets.

Dataset	Metrix	Signed Network Embedding		Signed Graph Neural Network		
		SiNE	Sign2vec	SGCN	SNEA	HSGAN
Bitcoin-A	AUC	0.781	0.790	0.801	0.816	0.891
	Micro-F1	0.825	0.824	0.864	0.874	0.928
	Macro-F1	0.655	0.665	0.706	0.743	0.791
	Binary-F1	0.895	0.892	0.915	0.927	0.959
Bitcoin-O	AUC	0.782	0.802	0.804	0.818	0.913
	Micro-F1	0.908	0.828	0.850	0.864	0.920
	Macro-F1	0.679	0.724	0.754	0.770	0.835
	Binary-F1	0.876	0.892	0.908	0.924	0.950
Slashdot	AUC	0.785	0.791	0.786	0.799	0.887
	Micro-F1	0.754	0.801	0.802	0.805	0.836
	Macro-F1	0.541	0.761	0.760	0.763	0.771
	Binary-F1	0.850	0.862	0.859	0.868	0.894
Epinion	AUC	0.831	0.859	0.849	0.861	0.925
	Micro-F1	0.858	0.854	0.872	0.887	0.903
	Macro-F1	0.671	0.785	0.800	0.816	0.819
	Binary-F1	0.902	0.912	0.920	0.933	0.941

### 4.5. Analysis and Discussion

In this subsection, we analyse the hyperparameters of the experiment, including how the effect of SiNP varies with the node representation dimension and signed-distance encoding vector dimension. We have conducted experiments on all three datasets, and because of space limitations we will only discuss the performance of the parameters on some of the dataset.

**Figure 3:**
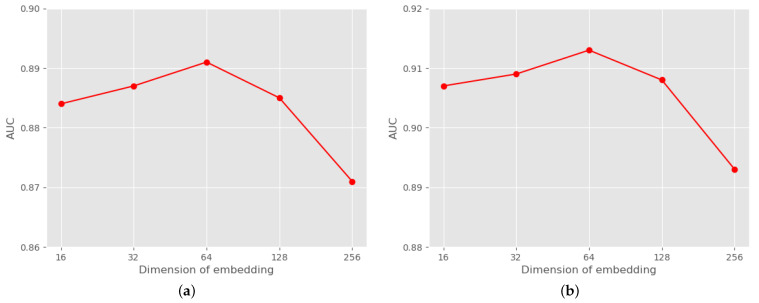
The dimension of final node embedding Z. (**a**) Bitcoin-Alpha. (**b**) Bitcoin-OTC.

The dimensionality of the node representation can directly affect the SiNP model performance. As shown in Figure 3, as the dimensionality of the node representation increases, the performance of the SiNP model first increases slowly, then remains constant, and finally will decrease slowly. This is because the SiNP model needs enough dimensions to store signed semantic and structural information, but too many dimensions can lead to redundancy and other problems caused by “overfitting”.

**Figure 4:**
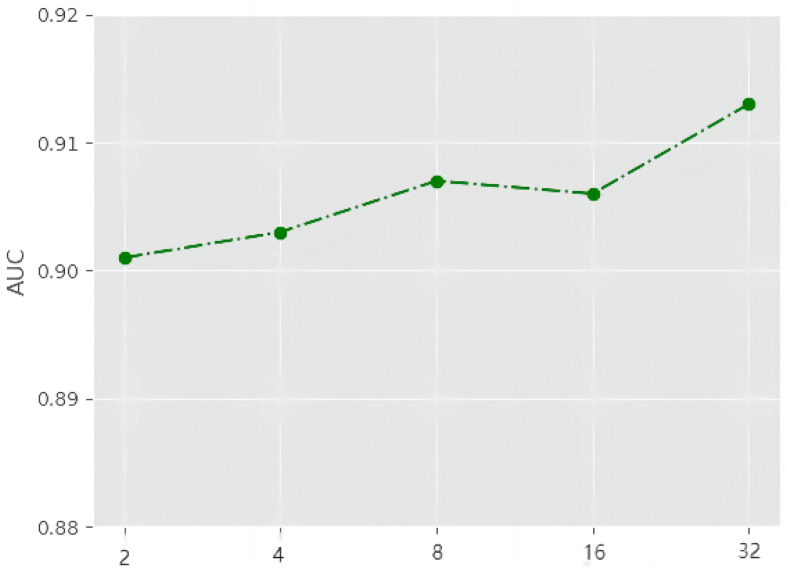
The dimension of signed-distance encoding vector.

To investigate the impact of the dimension of signed-distance encoding vector in our proposed SiNP framework, we further evaluated the efficacy of the SiNP framework with varying dimensions of the vector. As shown in Figure 4, the performance of the proposed SiNP framework tends to improve as the dimension increases, but from the finding, the increase is very limited. On the other hand, a large dimension can make the training process more stable.

### 4.6. Embedding Visualization

**Figure 5:**
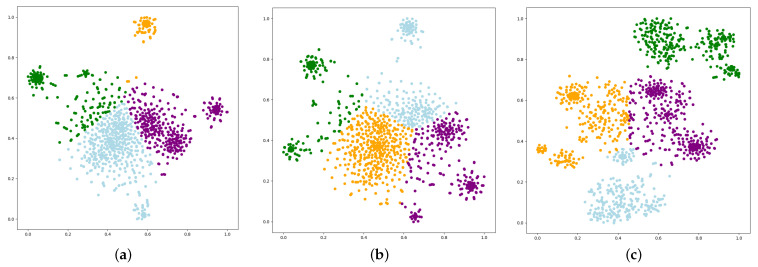
The visualization of the learned embeddings by SGNN baselines and the proposed SiNP on the Bitcoin-Alpha dataset. (**a**) SGCN. (**b**) SNEA. (**c**) SiNP.

To verify the effect of personalised features on the quality of node embedding, we performed a node embedding vector visualisation on the Bitcoin-Alpha dataset using the k-means algorithm with a category of 4 as shown in Figure 5. The node embeddings of the baseline algorithms SGCN, SNEA were taken out and visualised in two dimensions before being passed to the classifier. The different colours represent the different node class and it can be clearly seen that the embedding vectors obtained by SiNP can better cluster the nodes of different class and the distance between similar nodes is smaller.

## 5. Conclusions

In this paper, we present a unique deep learning based signed graph neural network with individual characteristics of the nodes. Specifically, we utilize a node signed property metric mechanism, which can encodes the individual characteristics of the nodes. Moreover, we also take into account the relations between node pairs and propose to aggregate information from balanced and unbalanced neighbors. In conclusion, our proposed SiNP model can accurately anticipate the sort of links that exist inside social networks and also be able to accurately forecast how negative associations are utilized within network systems, which helps to influence the design of social computing applications. The proposed model can be able to infer the underlying attitudes of users based on the data from the network and may be used to better recommend friends or favorite items to users in apps on social media. The findings reveal that our methodology was significantly more accurate, bolstering the legitimacy of the proposed method. SiNP improves the quality of the embedding vector only from the perspective of the node’s own characteristics, and in the future, we can consider fusing global or local information to see if it can be good for signed link prediction.

## Figures and Tables

**Table 1:** Basic information of the datasets.

Datasets	Nodes	Positive Links	Negative Links
Bitcoin-Alpha	3783	22,650	1536
Bitcoin-OTC	5881	32,029	3563
Slashdot	82,140	425,072	124,130
Epinion	131,828	717,667	123,705

## Data Availability

Not applicable.
